# Three‐dimensional computed tomography‐based comparison reveals divergent patellar and tibial tubercle behaviour between lateral closed‐wedge and medial open‐wedge high tibial osteotomy

**DOI:** 10.1002/jeo2.70884

**Published:** 2026-08-06

**Authors:** Yuta Tachibana, Kunihiko Hiramatsu, Kazutaka Kinugasa, Yuzo Yamada, Tomoya Nishikawa, Ayaka Tanaka, Norimasa Nakamura, Tomoki Ohori, Hiroyuki Tanaka, Seiji Okada, Yoshinari Tanaka

**Affiliations:** ^1^ Department of Orthopaedic Sports Medicine Osaka Rosai Hospital Sakai Osaka Japan; ^2^ Department of Orthopaedic Surgery Tamai Hospital Osaka Japan; ^3^ Department of Orthopaedic Surgery Yao Municipal Hospital Osaka Japan; ^4^ Department of Orthopaedic Surgery The University of Osaka Graduate School of Medicine Osaka Japan; ^5^ Institute for Medical Science in Sports Osaka Health Science University Osaka Japan; ^6^ Department of Food and Nutrition Osaka Metropolitan University Osaka Japan

**Keywords:** alignment, high tibial osteotomy, patellofemoral joint, three‐dimensional analysis, tibial tubercle

## Abstract

**Purpose:**

This study aimed to compare three‐dimensional patellar and tibial positional changes after medial open‐wedge high tibial osteotomy (MOWHTO) and lateral closed‐wedge high tibial osteotomy (LCWHTO) using computed tomography.

**Methods:**

This retrospective comparative cohort study included 18 patients who underwent high tibial osteotomy for medial knee osteoarthritis (9 MOWHTO, 9 LCWHTO). LCWHTO was selected for patients with patellofemoral joint degeneration, patellofemoral cartilage injury, anterior knee pain or correction angle ≥10°; MOWHTO was selected for the remaining patients. Full‐length lower‐limb computed tomography scans were obtained preoperatively and at 1 year postoperatively. Displacement and rotation of the patella and distal tibial fragment were quantified in the anterior–posterior, medial–lateral and proximal–distal directions and in the internal–external, varus–valgus and flexion–extension rotations and compared between groups.

**Results:**

In the MOWHTO group, the patella shifted distally (1.2 ± 1.9 mm) with internal (3.0° ± 1.9°) and valgus (1.9° ± 1.5°) rotation, whereas in the LCWHTO group it shifted proximally (1.6 ± 1.9 mm) with minimal rotation (proximal–distal displacement, *p* = 0.017; internal–external rotation, *p* < 0.001). The distal tibial fragment moved distally (3.5 ± 1.5 mm) with valgus rotation (8.1° ± 2.2°) in the MOWHTO group, but anteriorly (1.3 ± 0.7 mm), medially (6.7 ± 2.3 mm) and proximally (5.1 ± 2.2 mm) with internal rotation (4.5° ± 3.3°) in the LCWHTO group (all translations, *p* < 0.001; internal–external and varus–valgus rotation, *p* < 0.01). Patellar distalization and internal rotation correlated with tibial fragment distalization only in the MOWHTO group.

**Conclusion:**

MOWHTO and LCWHTO produced distinctly different three‐dimensional positional changes of the patella and distal tibial fragment. These positional findings provide a mechanistic basis for individualised surgical decision‐making, but their direct impact on PF cartilage status and long‐term outcomes requires confirmation in future studies.

**Level of Evidence:**

Level III, retrospective comparative cohort study.

**Abbreviations**
3D: three‐dimensional
AP: anterior–posterior
BMI: body mass index
CT: computed tomography
FE: flexion–extension
FPHI: femoral patellar height index
FTA: femorotibial angle
HKA: hip–knee–ankle angle
HTO: high tibial osteotomy
IE: internal–external
IRB: Institutional Review Board
JLCA: joint line convergence angle
KOOS: knee injury and osteoarthritis outcome score
LCWHTO: lateral closed‐wedge high tibial osteotomy
LDFA: lateral distal femoral angle
LPTA: lateral patellar tilting angle
LRR: lateral retinaculum release
MKI: Miura–Kawamura index
ML: medial–lateral
MOWHTO: medial open‐wedge high tibial osteotomy
MPTA: medial proximal tibial angle
OA: osteoarthritis
PD: proximal–distal
PF: patellofemoral
PTS: posterior tibial slope
VV: varus–valgus
%MA: percentage of mechanical axis

## INTRODUCTION

High tibial osteotomy (HTO) is a well‐established joint‐preserving surgical procedure for treating medial knee osteoarthritis (OA), particularly in younger and active patients with varus malalignment and preserved range of motion. Medial open‐wedge HTO (MOWHTO) and lateral closed‐wedge HTO (LCWHTO) are the most widely used procedures [28, 29]. The two procedures differ technically: MOWHTO involves a medial opening of the proximal tibia without requiring fibular osteotomy, whereas LCWHTO typically involves resection of a lateral tibial wedge with management of the proximal tibiofibular joint or fibular shaft [28, 29]. MOWHTO has been reported to induce distalization of the distal tibial fragment, including the tibial tubercle, thereby lowering patellar height, increasing patellofemoral (PF) joint contact pressure and potentially accelerating PF joint degeneration [9, 21, 26, 30]. In contrast, LCWHTO tends to proximalize the distal tibial fragment [29]. Several radiographic comparative studies have shown that LCWHTO has a relatively more favourable effect on PF joint parameters than MOWHTO. Ishimatsu et al. reported that compared with MOWHTO, hybrid LCWHTO improved PF joint congruity, including patellar height, lateral joint space and congruence angle at a minimum 5‐year follow‐up [9]. Otsuki et al. similarly demonstrated that LCWHTO preserved patellar height and significantly reduced the tibial tuberosity–trochlear groove (TT–TG) distance, whereas MOWHTO decreased patellar height without altering the TT–TG distance [21]. However, despite these radiographic differences, mid‐term clinical outcomes did not differ between the two procedures in either study, suggesting that the clinical implications of these PF changes remain unclear [9, 21]. Mechanistically, HTO induces three‐dimensional (3D) positional changes, including both translation and rotation, of the distal tibial fragment and the patella that cannot be fully characterised by conventional two‐dimensional plain radiographs or single‐plane computed tomography (CT) assessment [3, 12, 13, 15, 24]. Among biomechanical studies, only Gaasbeek et al. have directly compared the 3D change of the postoperative PF joint between MOWHTO and LCWHTO, and that study was performed in a cadaveric setting [5].

Therefore, this study aimed to evaluate 3D positional changes in the patella and tibia after MOWHTO and LCWHTO using CT images. We hypothesised that 3D positional changes of the patella and distal tibial fragment would differ significantly between MOWHTO and LCWHTO.

## METHODS

### Study design

This study was approved by the Institutional Review Board of Osaka Rosai Hospital (Approval No. 2022‐69) and was conducted in accordance with the principles of the Declaration of Helsinki. Written informed consent was obtained from all patients for participation. This was a retrospective comparative cohort including patients who underwent HTO for symptomatic medial knee OA with the Kellgren‐Lawrence grade I, II or III, unresponsive to ≥3 months of conservative treatment between April 2019 and December 2022. A total of 42 consecutive patients were enrolled in this study. The inclusion criteria included patients who had medial knee OA with tibial varus malalignment and underwent preoperative full‐length lower‐limb CT imaging. The exclusion criteria included ligament insufficiency, concomitant procedures affecting PF mechanics (e.g., lateral retinacular release), concomitant meniscal or cartilage repair and distal tibial tuberosity osteotomy. The choice between the two procedures was based on predefined indication criteria: LCWHTO was selected for patients with PF joint degeneration, PF cartilage injury, anterior knee pain or a planned correction angle ≥10°, whereas MOWHTO was selected for the remaining patients who met none of these criteria.

### Surgical procedures

Diagnostic arthroscopy was performed for all patients before the surgery. Meniscal, synovial or cartilage lesions that could cause mechanical symptoms were debrided as needed.

MOWHTO was performed using the standardised biplanar technique described by Takeuchi et al. [28]. The pes anserinus and superficial medial collateral ligament were partially detached. A transverse cut was made approximately 40 mm distal to the medial tibial plateau, parallel to the native posterior tibial slope (PTS), with care to preserve the hinge on the lateral side. Subsequently, a flange cut was made proximal to the tibial tuberosity, preserving a bone thickness of 10 mm, while the distal tibial bone fragment included the tibial tuberosity. Furthermore, the medial gap was opened gradually under fluoroscopic guidance according to preoperative planning, targeting a mechanical axis at 62.5% of the tibial plateau width [4]. The osteotomy gap was filled with two or three blocks of β‐tricalcium phosphate (OSferion 60; Olympus TerumoBiomaterials Corp.). The number of blocks was determined by the anteroposterior length of the transverse osteotomy plane, that is, the distance from the posterior margin of the flange cut to the posterior tibial cortex. The blocks were inserted sequentially from posterior to anterior so that the entire depth of the osteotomy plane was supported, and each block was shaped such that the anterior height was 1 mm shorter than the posterior height. Consequently, the anterior gap was at least 2 mm smaller than the posterior gap, in accordance with our institutional technique to avoid a symmetrical gap ratio and to minimise an increase in PTS [31]. A locking plate (TriS Medial HTO Plate System; Olympus TerumoBiomaterials Corp.) was then used for fixation.

LCWHTO was performed using the hybrid technique reported by Takeuchi et al. [29]. First, a fibular midshaft oblique osteotomy of approximately 30° was performed. Two 2.0 mm drill holes were created adjacent to the osteotomy, and a No. 2 polyester suture (FiberWire; Arthrex) was passed through them for later ligation [34]. Partial release of the anterior compartment fascia was performed. Subsequently, a flange cut was made proximal to the tibial tuberosity, preserving a bone thickness of 10 mm, while the distal tibial bone fragment included the tibial tuberosity. A transverse cut was created from approximately 40 mm distal to the lateral tibial plateau toward a point 15 mm distal to the medial joint line, with a hinge point where the transverse cut divided the tibia in a ratio of 2:1. The lateral wedged bone block was then removed, and complete osteotomy was achieved to the medial cortex. The proximal and distal tibial fragments were then reduced manually until lateral cortical contact was achieved. Fixation was performed with a lateral locking plate (TriS Lateral HTO Plate System; Olympus TerumoBiomaterials Corp.) with a supportive medial locking plate (AxSOS 3 Ti Proximal Tibia Plating System; Stryker Corp. or TriS Small HTO Plate System; Olympus TerumoBiomaterials Corp) Finally, the pre‐placed polyester suture was tightened to restore fibular compression.

### Postoperative rehabilitation

Postoperative rehabilitation was standardised for both groups. Range‐of‐motion exercises were initiated after 1 week of knee bracing, as tolerated by pain. Partial weight‐bearing was allowed at 2 weeks after MOWHTO and at 3 weeks after LCWHTO. Full weight‐bearing was allowed at 4 weeks after MOWHTO and at 5 weeks after LCWHTO. Heavy labour and sports activities were allowed at 3–4 months postoperatively after bone union was radiographically confirmed. The weight‐bearing protocol was delayed by one week in the LCWHTO group compared with the MOWHTO group because LCWHTO involves a complete osteotomy reaching the medial cortex and a concomitant fibular osteotomy, and the slower progression was intended to minimise the risk of loss of correction.

### CT assessment

Full‐length lower‐limb CT scans were obtained preoperatively, 2 days postoperatively and 1 year postoperatively using a 320‐detector row CT scanner (Aquilion Prime SP; Canon Medical Systems). The preoperative and 1‐year postoperative scans were used for the 3D positional change analysis, whereas the 2‐day postoperative scans were used to measure the hinge axis and flange angle before bone remodelling. Scans were performed with the patient in the supine position, knees fully extended and quadriceps relaxed to minimise muscle‐induced bias in patellar tracking. The Digital Imaging and Communications in Medicine data were imported into a 3D processing workstation, and bone surface models were generated using stereolithography‐based algorithms.

#### 3D change in the PF relationship

**Figure 1:**
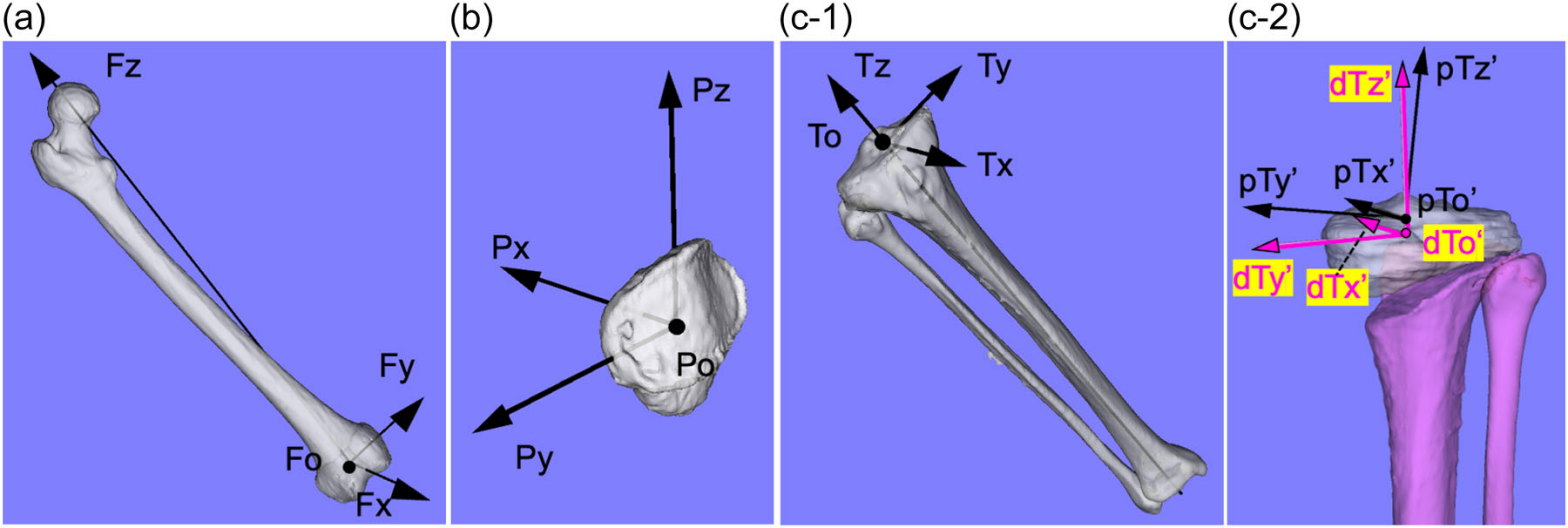
Coordinate axes (a) femur, (b) patella, (c‐1) preoperative tibia, (c‐2) postoperative tibia (seen from the posterior aspect) (a) The transepicondylar axis (TEA) was defined as the line between the medial and lateral epicondyles. The femoral origin (Fo) was defined as the midpoint of the TEA. The centre of the femoral head was determined by fitting a sphere to 100 surface points across the articular surface. The femoral proximal–distal axis (Fz) was created between Fo and the centre of the femoral head. The femoral XY plane was defined as the plane perpendicular to Fz and passing through Fo. The femoral medial–lateral axis (Fy) was created by projecting the TEA to the femoral XY plane. The femoral anterior–posterior axis (Fx) was the cross‐product of Fy and Fz. (b) The patellar origin (Po) was defined as the centroid of the patellar 3D model. A line connecting the most proximal and most distal points of the vertical posterior ridge line was created, and the patellar *Z*‐axis (Pz) was defined as the line parallel to this ridge and passing through Po. The patellar *Y*‐axis (Py) was defined as the line perpendicular to Pz and passing through the medial and lateral patellar borders. The patellar *X*‐axis (Px) was the cross‐product of Py and Pz. (c‐1) The tibial *Z*‐axis (Tz) was defined as the line connecting the tibial origin (To)—defined as the midpoint between the centres of the anterior and posterior cruciate ligament insertions—and the talar dome centre. A plane perpendicular to Tz and passing through To was created, and the tibial *X*‐axis (Tx) was defined as the line connecting the centres of the anterior and posterior cruciate ligament insertions projected onto this plane. The tibial *Y*‐axis (Ty) was the cross‐product of Tz and Tx. Fo, femoral origin; Fx/y/z, femoral x/y/z‐axis; Po, patellar origin; Px/y/z, patellar x/y/z‐axis; To: tibial origin; Tx/y/z, tibial x/y/z‐axis; pTo', proximal tibial origin (postoperatively); pTx'/y'/z', proximal tibial x/y/z‐axis (postoperatively); dTo', distal tibial origin (postoperatively); dTx'/y'/z', distal tibial x/y/z‐axis (postoperatively).

The femoral coordinate system was based on the centre of the femoral head and the medial/lateral epicondyles [16] (Figure 1a). The patellar coordinate system was based on the most proximal and most distal points of the vertical posterior ridge line and the medial and lateral patellar borders (Figure 1b) [22, 33]. The postoperative 1‐year femoral and patellar coordinate axes were obtained by applying the translational and rotational matrices, calculated by performing surface registration to superimpose the preoperative femoral and patellar models onto their respective postoperative 1‐year models [27].

**Figure 2:**
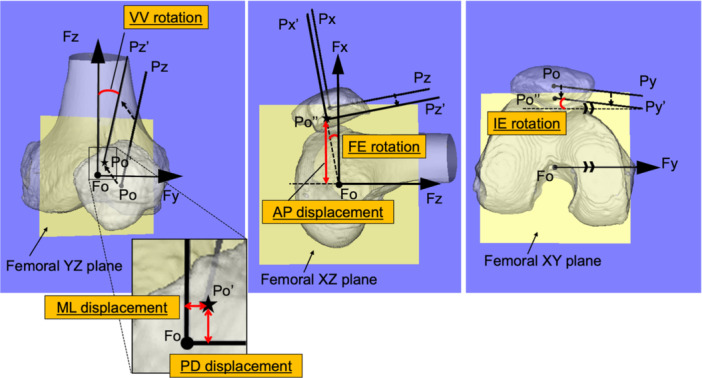
Patellofemoral relationship: displacement and rotation of the patella relative to the femoral coordinate system. Fo, femoral origin; Fx/y/z, femoral x/y/z‐axis; Po, patellar origin; Px/y/z, patellar x/y/z‐axis; Po'/Po”, patellar origin projected onto femoral XZ/XY plane; Px'/z', patellar *x*/*z*‐axis projected onto femoral XY plane; Py', patellar *Y*‐axis projected onto femoral XY plane; AP, anterior–posterior; ML, medial–lateral; PD, proximal–distal; IE, internal–external; VV, varus–valgus; FE, flexion–extension.

PF relationships were quantified as the displacement and rotation of the patella relative to the femoral coordinate system (Figure 2). Anterior–posterior (AP) displacement was defined as the distance from Po projected onto the femoral XZ plane (Po”) relative to Fz. Medial–lateral (ML) and proximal–distal (PD) displacement was defined as the distance from Po projected onto the femoral YZ plane (Po') relative to Fz and Fy, respectively. Internal–external (IE) rotation was defined as the angle between Py projected onto the femoral XZ plane and Fx. Varus–valgus (VV) rotation was defined as the angle between Pz projected onto the femoral YZ plane and Fz. Flexion–extension (FE) rotation was defined as the angle between Px projected onto the femoral XZ plane and Fx. Positive values indicated anterior, medial or proximal displacement and internal, varus or flexion rotation, respectively. Additionally, external rotation of the patella indicated an increase in patellar lateral tilt while internal rotation indicated a reduction in patellar lateral tilt. The measurement technique demonstrated excellent intra and interobserver reliability in previous studies (intraclass correlation coefficient >0.85) [27]. Changes in each parameter were evaluated from preoperative to 1‐year postoperative timepoint within each group and compared between the two groups.

#### 3D tibial change (distal fragment)

The patellar position is strongly influenced by the tibial tubercle position. Therefore, 3D positional changes in the distal tibial fragment, including the tibial tubercle, were evaluated relative to the proximal tibial fragment using the same AP/ML/PD (mm) and IE/VV/FE (°) system. The tibial coordinate system was created using the preoperative model according to a previously published method [16] (Figures 1c–1). The coordinate axes of the proximal and distal tibial fragments relative to the osteotomy plane were obtained postoperatively using the same method described above, and the translational and rotational differences of the distal fragment relative to the proximal fragment were calculated (Figures 1c‐2). Furthermore, correlation analyses between changes in patellar position and changes in distal tibial fragment position were performed separately for each group.

#### Knee flexion angle

Changes in knee flexion angle from preoperative to postoperative time points can affect the patellar position. Therefore, the knee flexion angle was calculated as the angle between the axis that Tz was projected onto the femoral XZ‐plane and Fz.

#### Hinge axis and flange angle

Hinge axis orientation can influence tibial rotation and PTS after HTO [2, 24, 31]. Therefore, axial hinge axis, sagittal hinge axis and flange angle were measured using 2‐day postoperative CT images according to the previous methods [2, 24, 31] (Figure S1). Comparisons of these parameters were performed between the two groups.

### Plain radiographic assessment

Standardised standing long‐leg anteroposterior radiographs, lateral radiographs and skyline views were obtained preoperatively and at the final follow‐up (≥ 24 months postoperatively). The hip–knee–ankle angle (HKA), femorotibial angle (FTA), medial proximal tibial angle (MPTA), lateral distal femoral angle (LDFA), joint line convergence angle (JLCA), percentage of mechanical axis (%MA) and femoral patellar height index (FPHI) [8] (Figure S2a) were evaluated on the standing long‐leg anteroposterior radiographs. Conventional radiographic assessments of patellar height have used anatomical landmarks, such as the tibial tubercle and the anterior edge of the tibial plateau [9, 10, 21]. However, since evaluation of patellar position relative to the femur is desirable because these landmarks are affected by HTO, we applied the FPHI [8], with high reproducibility. The FPHI uses the width of the femoral condyle to normalise the measurement, providing a reliable femur‐referenced index that is not distorted by morphological changes in the proximal tibia following osteotomy (Figure S2a). The Miura–Kawamura index (MKI) [17] Figure S2b), and PTS [32] were assessed on the lateral radiographs. The lateral patellar tilting angle (LPTA) [21] (Figure S2c) was evaluated on the skyline view. Postoperative changes were calculated and compared between the groups.

#### Clinical outcomes

Clinical outcomes were evaluated using the knee injury and osteoarthritis outcome score (KOOS) at 2 years postoperatively. Each subscale was compared between the two groups.

### Statistical analysis

All statistical analyses were performed using JMP Student Edition version 18.2.2 (SAS Institute Inc.). A power analysis with a power of 0.8 and a value of 0.05 indicated that a minimum of six patients in each group was required for comparing the PD displacement in the 3D PF relationship change between the two groups; a 2.5‐mm difference with a pooled standard deviation of ±1.5 mm. Likewise, a minimum of six patients in each group was required for comparing the IE rotation in 3D change of the distal tibial fragment between the two groups; a 5.1° difference with a pooled standard deviation of ±1.7°. Within‐group comparisons were performed using the Wilcoxon signed‐rank test. Between‐group comparisons were performed using Fisher's exact test for categorical variables and the Wilcoxon rank‐sum test for continuous variables. Correlation analysis between changes in patellar position and changes in distal tibial fragment position was performed using Spearman's rank correlation coefficient. For key comparisons, 95% confidence intervals are presented, and effect sizes for between‐group comparisons were calculated as *r* = |*Z* | /√*N*. A *p*‐value < 0.05 indicated statistical significance.

## RESULTS

### Patient demographics

**Figure 3:**
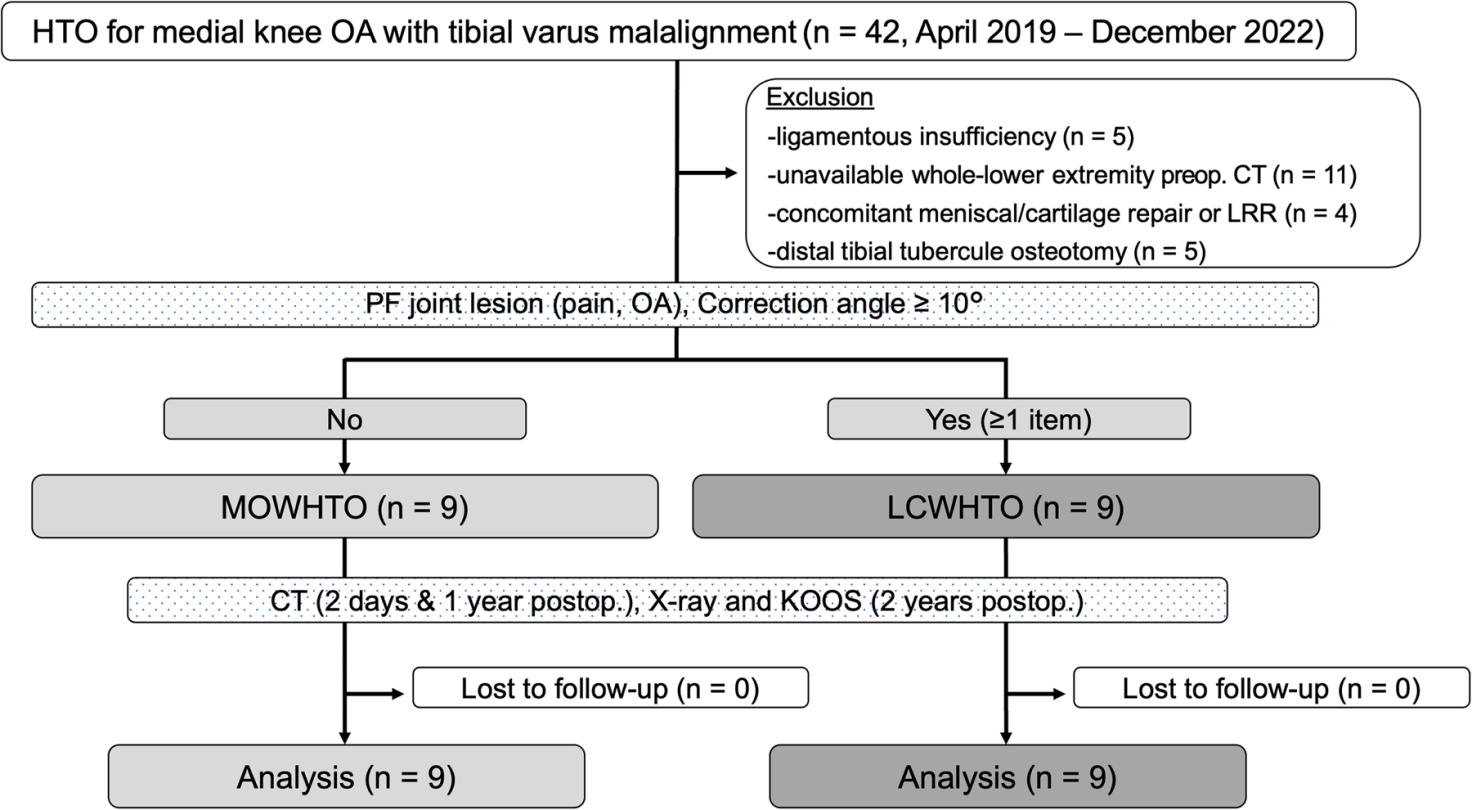
Flowchart of the study population. CT, computed tomography; HTO, high tibial osteotomy; KOOS, Knee Injury and Osteoarthritis Outcome Score; LCW, lateral closed wedge; LRR, lateral retinaculum release; MOW, medial open‐wedge; OA, osteoarthritis; PF, patellofemoral; postop., postoperatively; preop., preoperatively.

Eighteen patients met the inclusion criteria and were included in this study: Nine underwent MOWHTO, and nine underwent LCWHTO. The study cohort is summarised in the flowchart shown in Figure 3. Postoperative CT examinations at 2 days and 1 year, 2‐year plain radiographs and clinical scores were obtained in all the patients, while no patient was lost to follow‐up (Figure 3). Table 1 shows the demographic characteristics of the patients. The number of men in the LCWHTO group was significantly higher than that in the MOWHTO group (*p* = 0.044). The LCWHTO group demonstrated greater preoperative varus alignment and larger correction angles than the MOWHTO group (*p* < 0.01). The other demographic characteristics were comparable between the two groups.

**Table 1:** Patient demographics and preoperative parameters.

Parameter	MOWHTO (*n* = 9)	LCWHTO (*n* = 9)	*p*‐value	*r*
Sex (male/female)	3/6	8/1	0.044*	—
Laterality (right/left)	4/5	4/5	1.000	—
Age (years)	56.3 ± 8.5 (49.4–63.2)	57.3 ± 7.5 (51.2–63.5)	0.659	0.11
BMI (kg/m^2^)	25.0 ± 3.0 (22.6–27.4)	27.4 ± 3.9 (24.2–30.6)	0.170	0.32
Follow–up (months)	49.2 ± 14.0 (38.6–60.1)	46.7 ± 8.4 (39.5–53.9)	0.494	0.16
MPTA (°)	84.2 ± 3.1 (81.8–86.6)	82.3 ± 1.9 (80.9–83.8)	0.283	0.25
HKA (°)	–5.1 ± 2.4 (–6.9 to –3.3)	–8.5 ± 1.4 (–9.6 to –7.5)	<0.001*	0.66
FTA (°)	179.0 ± 1.8 (177.6–180.4)	182.2 ± 1.4 (181.1–183.3)	0.002*	0.72
JLCA (°)	2.1 ± 1.4 (1.1–3.2)	3.3 ± 1.8 (1.9–4.7)	0.154	0.33
LDFA (°)	87.0 ± 1.2 (86.1–87.9)	87.7 ± 0.9 (87.0–88.3)	0.123	0.35
%MA (%)	27.0 ± 10.0 (19.3–34.7)	12.0 ± 5.6 (7.6–16.3)	0.003*	0.68
Correction angle (°)	8.3 ± 1.7 (7.0–9.7)	11.4 ± 1.0 (10.7–12.2)	0.003*	0.70
PTS (°)	7.1 ± 2.4 (5.3–8.9)	9.5 ± 2.4 (7.6–11.3)	0.074	0.43
LPTA (°)	9.3 ± 2.5 (7.4–11.2)	7.8 ± 3.2 (5.4–10.2)	0.228	0.28
FPHI	0.65 ± 0.07 (0.60–0.70)	0.65 ± 0.08 (0.59–0.71)	0.574	0.14
MKI	0.70 ± 0.13 (0.60–0.79)	0.70 ± 0.14 (0.59–0.80)	0.834	0.05
K‐L grade (0/I/II/III/IV)	0/3/5/1/0	0/2/4/3/0	0.362	—

*Note*: Values are presented as mean ± SD (95% CI). The effect size r was calculated from the Wilcoxon rank–sum test (r = |Z | /√N). For categorical variables, *p* values were calculated by Fisher's exact test and effect sizes are not applicable.

Abbreviations: %MA, percentage of mechanical axis; BMI, body mass index; FPHI, femoral patellar height index; FTA, femorotibial angle; HKA, hip–knee–ankle angle; JLCA, joint line convergence angle; KL grade, Kellgren–Lawrence grade; LCWHTO, lateral closed–wedge high tibial osteotomy; LDFA, lateral distal femoral angle; LPTA, lateral patellar tilting angle; MKI, Miura–Kawamura index; MOWHTO, medial open–wedge high tibial osteotomy; MPTA, medial proximal tibial angle; PTS, posterior tibial slope.

* *p* < 0.05.

### CT assessment

#### 3D change in the PF relationship

##### Displacement

**Figure 4:**
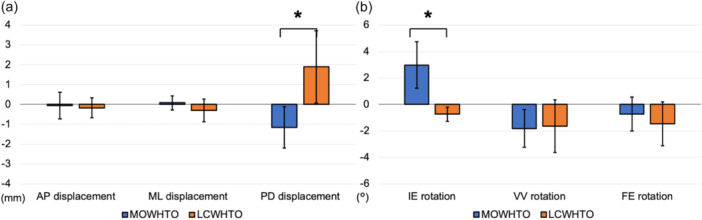
3D changes in the PF relationship. (a) Displacement and (b) rotation. AP, anterior–posterior; FE, flexion–extension; IE, internal–external; LCWHTO, lateral closed‐wedge high tibial osteotomy; ML, medial–lateral; MOWHTO, medial open‐wedge high tibial osteotomy; PD, proximal–distal; VV, varus–valgus. Positive values indicate anterior, medial or proximal displacement and internal, varus or flexion rotation of the patella. *p < 0.05 (Wilcoxon rank‐sum test).

The patella moved anteriorly by 0.1 ± 0.4 mm, laterally by 0.1 ± 0.7 mm and distally by 1.2 ± 1.9 mm after MOWHTO, whereas it moved posteriorly by 0.3 ± 0.6 mm, laterally by 0.2 ± 0.5 mm and proximally by 1.6 ± 1.9 mm after LCWHTO. A significant within‐group change in PD displacement was observed in both groups (*p* = 0.046 and 0.046). Between‐group comparisons showed a significant difference in PD displacement (*p* = 0.013) (Figure 4a).

##### Rotation

The patella demonstrated 3.0° ± 1.9° internal rotation, 1.9° ± 1.5° valgus rotation and 0.7° ± 1.4° extension rotation after MOWHTO. Internal and valgus rotation were significantly different from preoperative values (*p* = 0.008 and 0.004). The patella demonstrated 0.7° ± 0.6° external rotation, 1.6° ± 2.0° valgus rotation and 1.5° ± 1.7° extension rotation after LCWHTO. No significant within‐group changes were observed. Between‐group comparison showed a significant difference in IE rotation (*p* < 0.001) (Figure 4b).

#### 3D change of distal tibial fragment

##### Displacement

**Figure 5:**
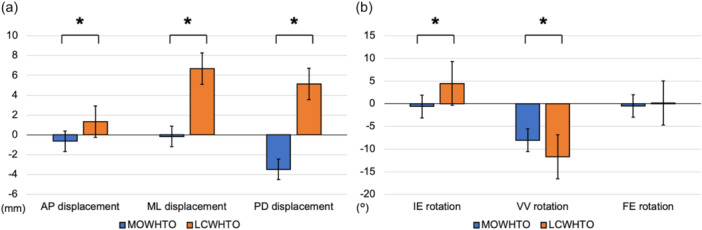
3D change in the distal tibial fragment relative to the proximal one. (a) Displacement and (b) rotation. AP, anterior–posterior; FE, flexion–extension; IE, internal–external; LCWHTO, lateral closed‐wedge high tibial osteotomy; ML, medial–lateral; MOWHTO, medial open‐wedge high tibial osteotomy; PD, proximal–distal; VV, varus–valgus. Positive values indicate anterior/medial/proximal displacement and internal/varus/flexion rotation of the distal tibial fragment. *p < 0.05 (Wilcoxon rank‐sum test).

The distal tibial fragment moved posteriorly by 0.6 ± 0.7 mm, laterally by 0.2 ± 1.6 mm and distally by 3.5 ± 1.5 mm after MOWHTO. A significant within‐group change in PD displacement was observed (*p* = 0.004). The distal fragment moved anteriorly by 1.3 ± 0.7 mm, medially by 6.7 ± 2.3 mm and proximally by 5.1 ± 2.2 mm after LCWHTO. Significant within‐group changes were observed in all parameters (*p* = 0.004 in all parameters). Between‐group comparisons showed significant differences in all translational parameters (*p* < 0.001) (Figure 5a).

##### Rotation

The distal tibial fragment demonstrated 0.6° ± 2.4° external rotation, 8.1° ± 2.2° valgus rotation and 0.5° ± 2.7° extension rotation after MOWHTO. A significant within‐group change in VV rotation was observed (*p* = 0.004). The distal tibial fragment demonstrated 4.5° ± 3.3° internal rotation, 11.7° ± 2.3° valgus rotation, and 0.2° ± 2.1° flexion rotation after LCWHTO. Significant within‐group changes were observed in IE and VV rotation (*p* = 0.004 and 0.004). Between‐group comparisons showed significant differences in IE and VV rotation (*p* = 0.008 and 0.008) (Figure 5b).

**Figure 6:**
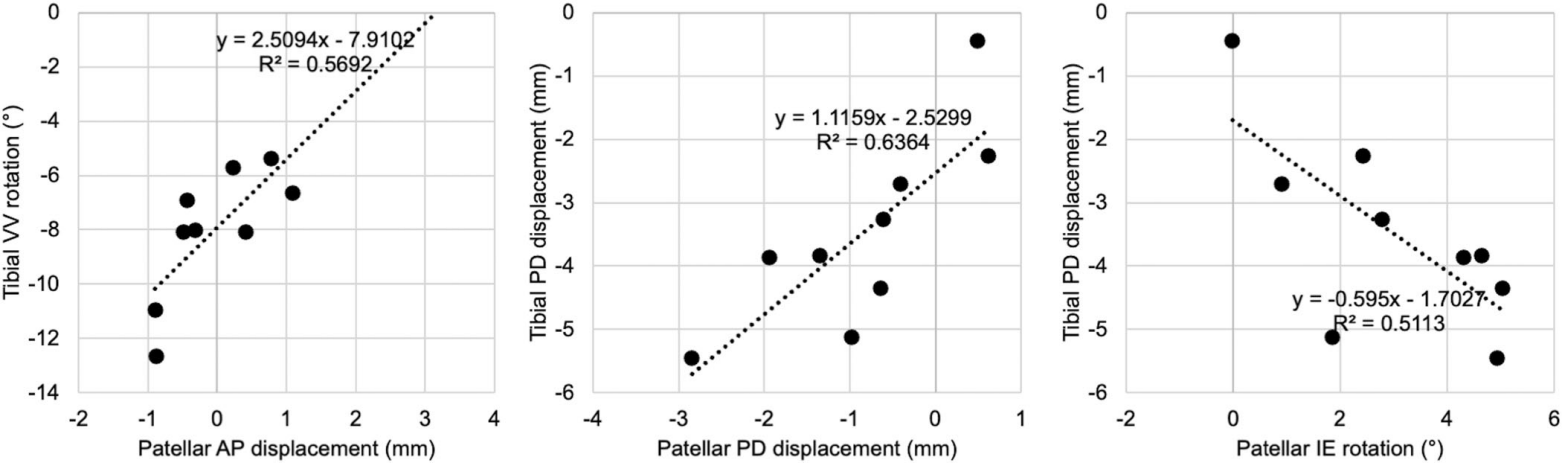
Correlation between tibial fragment motion and patellar motion in the MOWHTO group. AP, anterior–posterior; IE, internal–external; MOWHTO, medial open‐wedge high tibial osteotomy; PD, proximal–distal; VV, varus–valgus. Positive values indicate anterior and proximal displacement and varus and internal rotation.

Correlation analysis between tibial fragment motion and patellar motion indicated significant correlations between patellar AP displacement and tibial VV rotation (*ρ* = 0.75, *p* = 0.013), between patellar PD displacement and tibial PD displacement (*ρ* = 0.80, *p* = 0.005), and between patellar IE rotation and tibial PD displacement (*ρ* = −0.72, *P* = 0.030) in the MOWHTO group (Figure 6). However, no significant correlations were observed in the LCWHTO group.

#### Knee flexion angle, hinge axis and flange axis

Minimal changes in knee flexion angle from preoperative to postoperative scans were observed in both groups, and no significant within‐ or between‐group differences were found. Similarly, the axial hinge axis, sagittal hinge axis and flange angle did not differ significantly between the two groups (Table S1).

### Plain radiographic assessment

The coronal alignment was successfully corrected postoperatively as planned in both groups. After MOWHTO, significant decreases were observed in FPHI (−0.03 ± 0.03, *p* = 0.027) and MKI (−0.08 ± 0.07, *p* = 0.027), indicating patellar distalization and decrease in LPTA (−3.7 ± 1.6°, *p* = 0.012) was observed. Conversely, after LCWHTO, significant increases in FPHI (+0.06 ± 0.07, *p* = 0.012) and MKI (+0.09 ± 0.10, *p* = 0.027), indicating patellar proximalization, and significant decreases in PTS (−1.4 ± 1.6°, *p* = 0.046) were observed. Comparisons of the postoperative values showed significant differences in FPHI (*p* = 0.013) and MKI (*p* = 0.033). Furthermore, significant differences in changes before and after surgery in all radiographic parameters, except for PTS, were observed between the two groups (Table 2).

**Table 2:** Postoperative radiographic parameters and change from baseline.

Parameter	Time	MOWHTO (*n* = 9)	LCWHTO (*n* = 9)	*p*‐value	*r*
MPTA (°)	Postoperative	92.3 ± 1.9 (90.8–93.9)	92.8 ± 2.1 (91.0–94.5)	0.749	0.07
	Change	8.1 ± 2.4 (6.1–10.1)^a^	10.4 ± 2.0 (8.8–12.1)^a^	0.040^b^	0.47
HKA (°)	Postoperative	3.1 ± 1.7 (1.8–4.5)	2.3 ± 1.8 (0.8–3.8)	0.504	0.16
	Change	8.2 ± 2.5 (6.2–10.2)^a^	10.9 ± 2.5 (8.7–13.0)^a^	0.044^b^	0.44
FTA (°)	Postoperative	171.2 ± 1.7 (169.8–172.6)	171.3 ± 1.9 (169.7–172.9)	0.816	0.05
	Change	–7.8 ± 2.1 (–9.5 to –6.1)^a^	–10.9 ± 2.5 (–12.9 to –8.8)^a^	0.033^b^	0.51
JLCA (°)	Postoperative	1.4 ± 1.1 (0.6–2.3)	0.7 ± 1.6 (–0.7 to 2.0)	0.372	0.21
	Change	–0.7 ± 1.1 (–1.5 to 0.2)	–2.7 ± 2.1 (–4.3 to –1.0)^a^	0.033^b^	0.50
%MA (%)	Postoperative	61.4 ± 5.7 (56.8–66.1)	62.0 ± 6.9 (56.4–67.7)	0.964	0.01
	Change	34.4 ± 9.1 (27.0–41.8)^a^	50.1 ± 9.4 (42.4–57.7)^a^	0.006^b^	0.63
FPHI	Postoperative	0.62 ± 0.05 (0.57–0.66)	0.70 ± 0.05 (0.66–0.74)	0.011^b^	0.59
	Change	–0.03 ± 0.03 (–0.06 to –0.01)^a^	0.06 ± 0.07 (–0.00 to 0.11)^a^	0.001^b^	0.72
PTS (°)	Postoperative	6.6 ± 3.3 (3.9–9.2)	8.0 ± 2.9 (5.6–10.4)	0.354	0.22
	Change	–0.6 ± 2.4 (–2.5 to 1.4)	–1.4 ± 1.6 (–2.7 to –0.2)^a^	0.214	0.28
MKI	Postoperative	0.62 ± 0.13 (0.51–0.73)	0.79 ± 0.18 (0.64–0.94)	0.033^b^	0.45
	Change	–0.08 ± 0.07 (–0.13 to –0.03)^a^	0.09 ± 0.10 (0.02–0.17)^a^	0.001^b^	0.76
LPTA (°)	Postoperative	5.6 ± 3.2 (3.0–8.2)	6.4 ± 2.7 (4.2–8.7)	0.789	0.07
	Change	–3.7 ± 1.6 (–4.9 to –2.5)^a^	–1.1 ± 1.3 (–2.6 to –0.1)	0.009^b^	0.59

*Note*: Values are presented as mean ± SD (95% CI). The effect size *r* was calculated from the Wilcoxon rank–sum test (*r* = |*Z* | /√*N*) for between–group comparisons of postoperative values and of pre to postoperative changes.

Abbreviations: %MA, percentage of mechanical axis; FPHI, femoral patellar height index; FTA, femorotibial angle; HKA, hip–knee–ankle angle; JLCA, joint line convergence angle; LCWHTO, lateral closed–wedge high tibial osteotomy; LPTA, lateral patellar tilting angle; MKI, Miura–Kawamura index; MOWHTO, medial open–wedge high tibial osteotomy; MPTA, medial proximal tibial angle; PTS, posterior tibial slope.

^a^ Significant within–group change from preoperative to postoperative (*p* < 0.05; Wilcoxon signed–rank test).

b Between–group *p* < 0.05 (Wilcoxon rank–sum test).

### Clinical outcomes

All subscales in the KOOS at 2 years postoperatively were comparable between the two groups (Table S2). No significant complications, including nonunion, loss of correction, hinge fracture requiring intervention, infection or postoperative extension or flexion loss ≥10°, were observed in either group.

## DISCUSSION

The principal finding of this study was that MOWHTO and LCWHTO produced markedly different 3D positional changes of both the patella and the distal tibial fragment. MOWHTO induced distal translation and internal rotation (reduction in lateral tilt) of the patella in association with distal migration of the distal tibial fragment, whereas LCWHTO induced proximal patellar migration with minimal rotational change in association with anterior, medial and proximal translation of the distal tibial fragment. To our knowledge, this is the first in vivo CT‐based comparison of 3D positional changes of both the patella and the distal tibial fragment between MOWHTO and LCWHTO.

**Figure 7:**
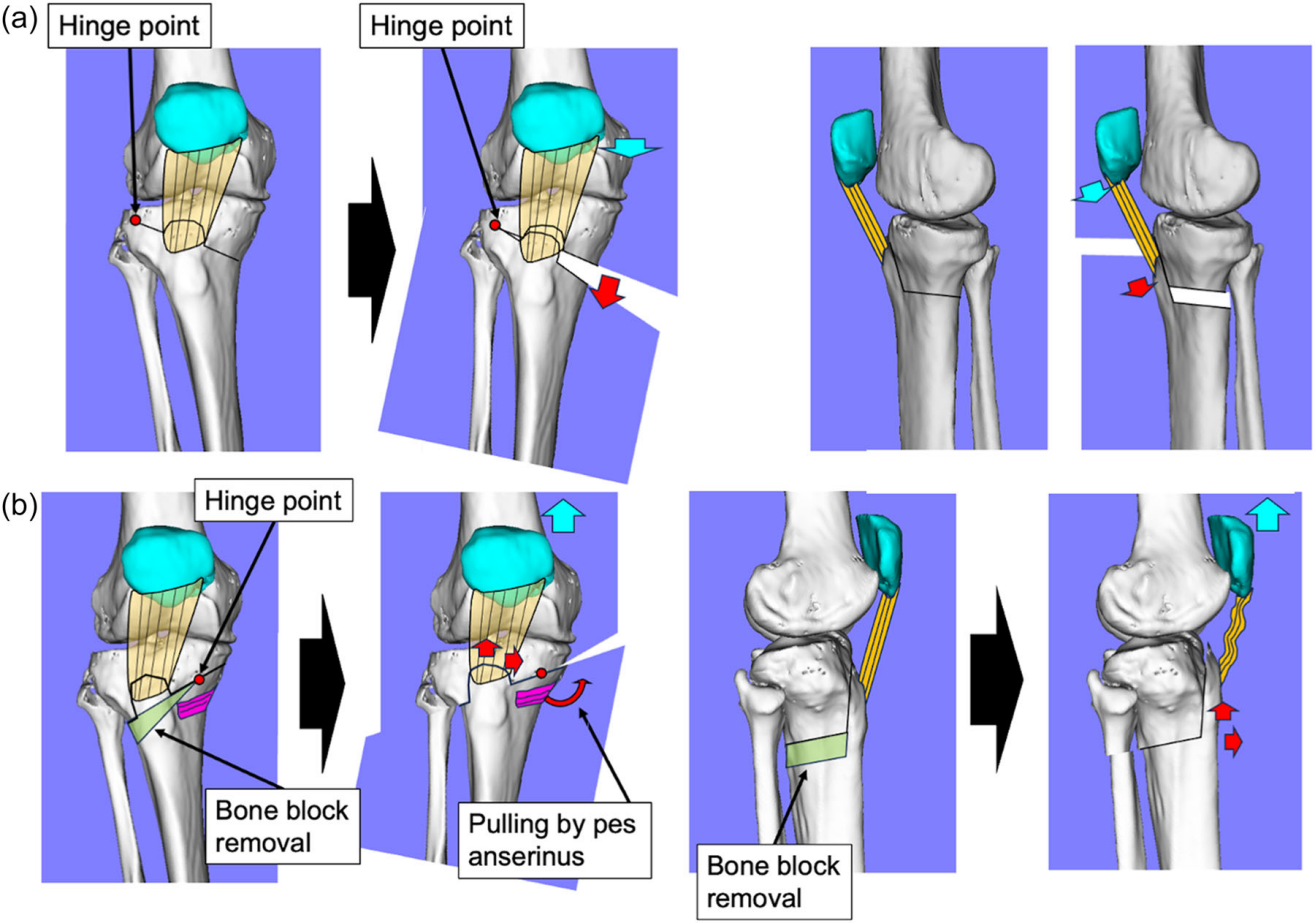
Schemas after high tibial osteotomies (a) After medial open‐wedge high tibial osteotomy: the distal tibial fragment, including the tibial tubercle, opens distally with a predominantly medial widening around the proximal lateral hinge, resulting in tibial tubercle lateralisation. (b) After lateral closed‐wedge high tibial osteotomy: the proximal, anterior and medial translation and internal rotation of the distal tibial fragment, including the tibial tuberosity, reduce the extensor mechanism.

The patella demonstrated not only distal translation but also internal and valgus rotation after MOWHTO. After MOWHTO, the distal tibial fragment, including the tibial tubercle, opens distally with a predominantly medial widening around the proximal lateral hinge, resulting in tibial tubercle lateralisation (Figure 7a). Numerous previous studies have shown that the patella translates distally, causing patella baja and medial‐dominant distal traction produces patellar valgus rotation [9, 10, 13].

In this study, the patella exhibited internal rotation, indicating reduced lateral tilt after MOWHTO. Kameda et al. reported that distal–lateral translation of the distal tibial fragment increases contact pressure between the patellar lateral articular surface and the lateral trochlear wall, thereby increasing reactive forces and producing patellar internal rotation [10]. Given that the patella is continuous with the tibial tubercle through the patellar tendon, PF alignment after HTO may be influenced by translational and rotational changes in the distal tibial fragment. Gaasbeek et al. in cadaveric studies reported increased patellar internal rotation with larger correction angles [5], consistent with our findings. Although no significant changes in patellar AP displacement from pre‐ to postoperative evaluations were observed in our study, a significant positive correlation was observed between tibial VV rotation and patellar AP displacement. Because the flange was formed at approximately 110° relative to the transverse osteotomy plane, the distal tibial fragment, including the tibial tubercle, moved distally and posteriorly after MOWHTO (Figure 7a). Therefore, as valgus correction increases, the patellar tendon may be tensioned along this vector, resulting in posterior patellar translation along the trochlear contour. Although some studies have shown that PF degeneration after MOWHTO does not necessarily affect clinical outcomes [6, 7], concerns remain about increased PF contact pressure [10, 14, 25]. Otakara et al. and Tanaka et al. reported an association between MPTA changes ≥9° or opening distances ≥13 mm and the progression of PF cartilage degeneration after MOWHTO [20, 30]. Therefore, in patients requiring large corrections or in those with concerning PF pathology or preexisting patella baja, alternative techniques such as LCWHTO, distal tibial tubercle osteotomy as part of MOWHTO or double‐level osteotomy may be considered. Several studies have shown that distal tibial tubercle osteotomy does not cause patellar baja or increase PF contact pressure [14] and provides better clinical outcomes [19] than the standard MOWHTO. However, a recent study warned of the risk of increased PTS, which can adversely affect bone union in the flange [1].

Compared with MOWHTO, relatively few studies have evaluated 3D positional and rotational changes in the patella after LCWHTO. The distal tibial fragment, including the tibial tubercle, exhibited significant anterior [12, 29], medial [13] and proximal translation [5, 13] and internal [15] and valgus rotation. These changes may be due to the flange geometry being angled approximately 110° from the transverse osteotomy plane, promoting proximal and anterior displacement of the tibial tubercle, and the fact that LCWHTO completely cuts the tibia from lateral to medial, thereby allowing the pes anserinus attached to the distal tibial fragment to exert medializing and internal rotational forces [13, 15] (Figure 7b) Anteromedialization tibial tubercle osteotomy has been reported to be effective for PF articular cartilage disorders without instability [23]. These positional changes observed after LCWHTO, particularly the medial translation of approximately 6.7 mm, may be clinically meaningful in patients with increased TT–TG distance, whereas the anterior translation of 1.3 mm is small and its clinical impact remains uncertain. Moreover, although recent studies have shown an association between a decreased sagittal TT–TG distance and PF chondral pathology [11, 18], the proximalization of the patella after LCWHTO may not be desirable in patients with pre‐existing patella alta, in whom the PF condition could potentially be worsened. Therefore, the surgical strategy should be individualised rather than uniformly recommending one procedure over the other. In this study, LCWHTO caused approximately 1° patellar rotational change in both CT (IE rotation) and radiograph (LPTA) evaluations, without any significances. These findings were consistent with previous studies [5, 21]. Regarding the correlations between patellar position and distal tibial fragment position after LCWHTO, no significant correlation of 3D positional changes was observed in this study, although no previous study has investigated this relationship. These findings may indicate relaxation of the extensor mechanism due to proximalization of the tibial tubercle, thereby reducing mechanical coupling between tibial and patellar rotation.

MOWHTO has been reported to potentially increase PTS depending on the hinge axis orientation and the geometry of the medial opening, whereas LCWHTO has been reported to either maintain or slightly decrease PTS [2, 9, 21, 24, 31]. In the present study, the within‐group change in PTS reached statistical significance only in the LCWHTO group (−1.4 ± 1.6°, *p* = 0.046), but the between‐group difference in PTS change was not significant (Table 2). This is likely attributable to our careful intraoperative attention to maintaining a hinge axis parallel to the native PTS and adjusting the gap ratio during MOWHTO.

This study has several limitations. First, the sample size was small. The power analysis was conducted for two primary endpoints (PD patellar displacement and IE rotation of the distal tibial fragment). Secondary comparisons in this study, including all other displacement and rotational parameters and clinical outcomes, were not formally powered. Therefore, nonsignificant findings for secondary endpoints should be interpreted with caution, as the risk of type II error cannot be excluded. Second, our CT‐based analysis quantified static, non‐weight‐bearing positional changes with the quadriceps relaxed in full extension. However, clinical PF degeneration is driven by dynamic, loaded kinematics during functional activities such as walking, squatting and stair climbing. Therefore, our findings may not fully reflect the in vivo functional behaviour of the patella, and further studies using dynamic imaging (e.g., dual‐fluoroscopy or motion‐capture) are needed to confirm whether the static positional changes observed in this study correspond to dynamic kinematic differences. Third, the two groups are not fully comparable because the surgical indications were different: LCWHTO was selected for patients with preoperative PF pathology or a larger correction angle (≥ 10°), and the LCWHTO group consequently demonstrated significantly greater preoperative varus deformity and larger correction angles than the MOWHTO group. These selection‐related factors could act as confounders, since a larger correction angle in MOWHTO is known to increase PF contact pressure. Fourth, the only clinical outcome available in this study, the KOOS at 2 years, did not show any between‐group difference. Given the small sample size and the relatively short follow‐up, this null finding should be acknowledged when interpreting our results, and whether the observed positional differences translate into clinically meaningful PF outcomes remains uncertain.

## CONCLUSION

MOWHTO and LCWHTO produced distinctly different 3D positional changes of the patella and distal tibial fragment. MOWHTO induced distalization and internal/valgus rotation of the patella with distal migration of the distal tibial fragment, whereas LCWHTO induced proximal patellar migration with minimal rotational change and anteromedial, proximal translation of the distal tibial fragment. These positional findings provide a mechanistic basis for individualised surgical decision‐making, but their direct impact on PF cartilage status and long‐term outcomes requires confirmation in future studies.

## AUTHOR CONTRIBUTIONS

All authors contributed to the study conception and design. Data collection was performed by Yuta Tachibana, Kunihiko Hiramatsu, Kazutaka Kinugasa, Yoshinari Tanaka and Yuzo Yamada. Three‐dimensional data analysis was performed by Yuta Tachibana. Statistical analysis was performed by Yuta Tachibana. The first draft of the manuscript was written by Yuta Tachibana, and all authors commented on previous versions of the manuscript. Supervision of the project was provided by Norimasa Nakamura, Tomoki Ohori, Hiroyuki Tanaka and Seiji Okada. All authors read and approved the final manuscript and agree to be accountable for all aspects of the work.

## FUNDING INFORMATION

The authors have no funding to report.

## CONFLICT OF INTEREST STATEMENT

The authors declare no conflicts of interest.

## ETHICS STATEMENT

This retrospective study involving human participants was performed in line with the principles of the Declaration of Helsinki. Approval was granted by the Institutional Review Board of Osaka Rosai Hospital (Approval No. 2022‐69; date of approval: 09/20/2022). All procedures performed in this study were in accordance with the ethical standards of the institutional and/or national research committee and with the 1964 Helsinki Declaration and its later amendments or comparable ethical standards. Written informed consent was obtained from all individual participants included in the study prior to enrollment. The authors affirm that human research participants provided informed consent for publication of all data and images included in this article; no identifying information is included.

## Supporting information

Supporting file 1.

Supporting file 2.

Supporting file 3.

Supporting file 4.

## Data Availability

The datasets generated and/or analysed during the current study are not publicly available due to patient privacy restrictions but are available from the corresponding author on reasonable request and subject to approval by the Institutional Review Board of Osaka Rosai Hospital.
