# Supplementary material for: Three‐dimensional computed tomography‐based comparison reveals divergent patellar and tibial tubercle behaviour between lateral closed‐wedge and medial open‐wedge high tibial osteotomy
Source: J Exp Orthop. 2026 Aug 6;13(3):e70884. doi: 10.1002/jeo2.70884 (PMC13446280; doi:10.1002/jeo2.70884)
Supplement: Supplementary file 1 [file JEO2-13-e70884-s003.pdf]

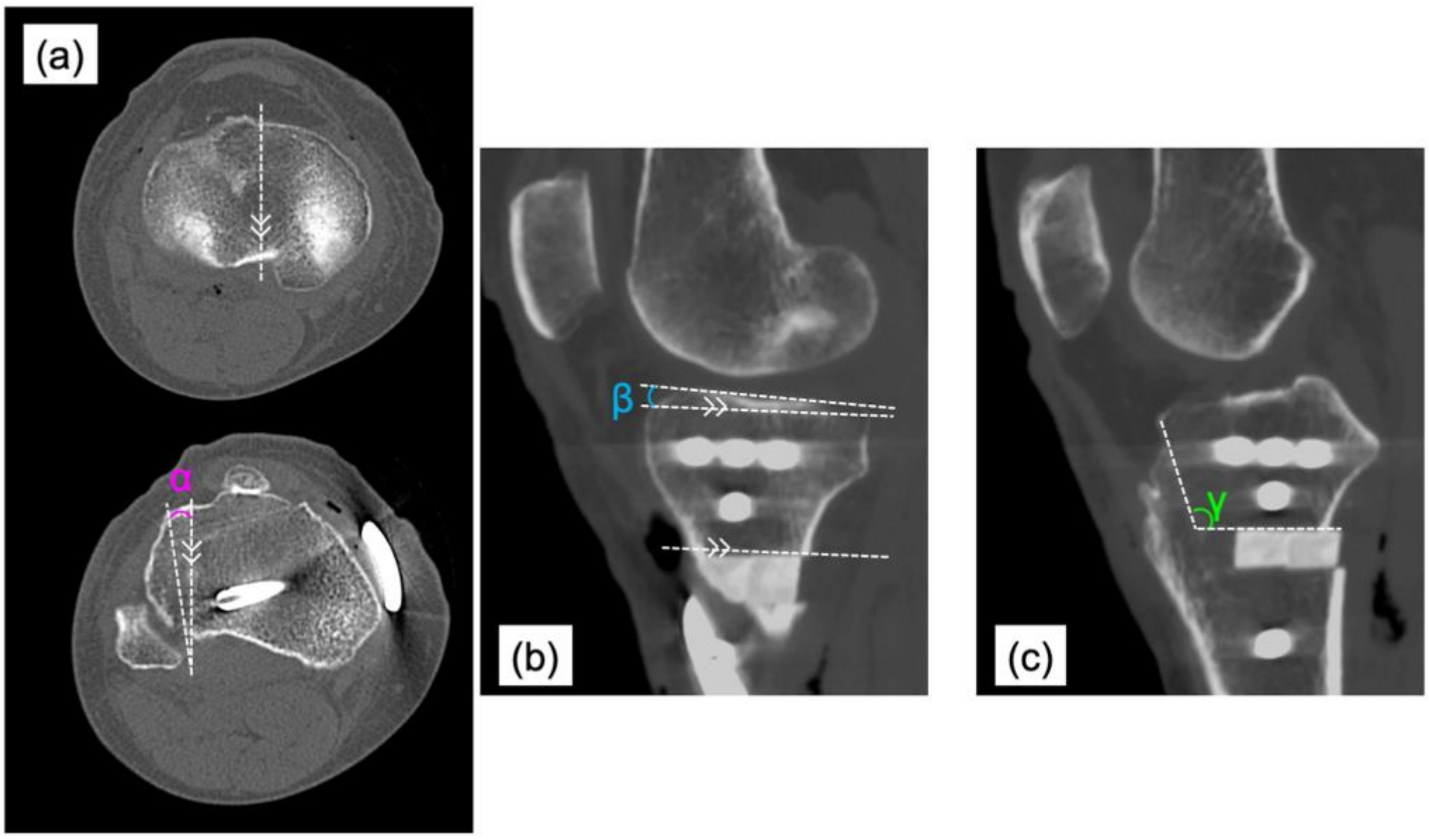

Figure S1 Hinge axis and flange angle

(a) Axial hinge axis (α) was defined as the angle between the hinge axis and the proximal tibial X-axis on the axial plane (positive value: external rotation). (b) Sagittal hinge axis (β) was defined as the angle between the transverse osteotomy plane and the slope of the medial tibial plateau (positive value: posterior inclination). (c) Flange angle (γ) was defined as the angle between the osteotomy flange and the transverse osteotomy plane (positive value: anterior inclination of the osteotomy flange).
