# Supplementary material for: Three‐dimensional computed tomography‐based comparison reveals divergent patellar and tibial tubercle behaviour between lateral closed‐wedge and medial open‐wedge high tibial osteotomy
Source: J Exp Orthop. 2026 Aug 6;13(3):e70884. doi: 10.1002/jeo2.70884 (PMC13446280; doi:10.1002/jeo2.70884)
Supplement: Supplementary file 2 [file JEO2-13-e70884-s004.pdf]

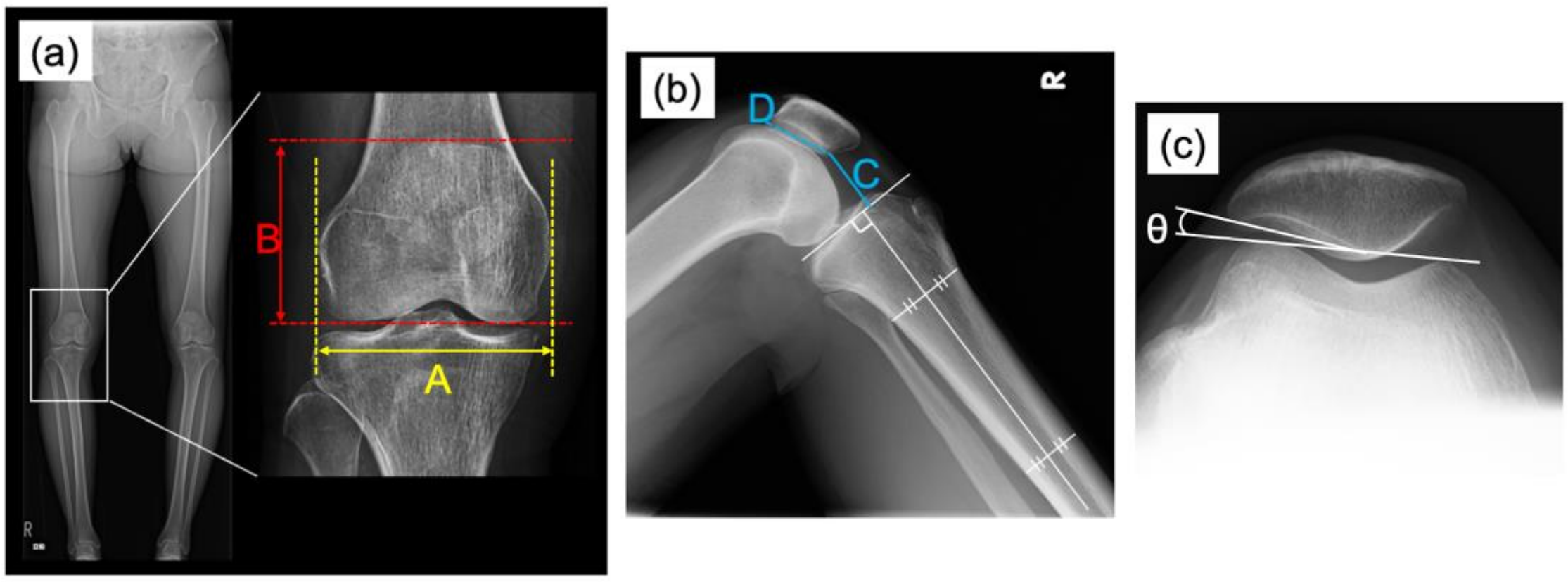

Figure S2 Patellar position relative to the femur on plain radiographs

(a) The femoral patellar height index (A/B), (b) the modified Miura–Kawamura index (C/D), and (c) the lateral patellar tilting angle (θ).
