# Supplementary material for: Three‐dimensional computed tomography‐based comparison reveals divergent patellar and tibial tubercle behaviour between lateral closed‐wedge and medial open‐wedge high tibial osteotomy
Source: J Exp Orthop. 2026 Aug 6;13(3):e70884. doi: 10.1002/jeo2.70884 (PMC13446280; doi:10.1002/jeo2.70884)
Supplement: Supplementary file 3 [file JEO2-13-e70884-s001.docx]

**Table S1. Knee flexion angle, hinge axis, and flange angle**

| **Parameter** | **MOWHTO (n = 9)** | **LCWHTO (n = 9)** | **P value** | ***r*** |
| --- | --- | --- | --- | --- |
| Knee flexion angle (°) | −0.2± 2.3 (−1.6 to 1.9) | 0.4 ± 2.4 (−1.6 to 2.3) | 0.124 | 0.37 |
| Axial hinge axis (°) | 10.5 ± 2.5 (8.6 to 12.4) | 8.1 ± 4.2 (4.9 to 11.2) | 0.134 | 0.36 |
| Sagittal hinge axis (°) | 6.2 ± 4.1 (3.0 to 9.4) | 4.8 ± 4.0 (1.7 to 7.8) | 0.764 | 0.07 |
| Flange angle (°) | 109.8 ± 7.5 (104.1 to 115.6) | 110.2 ± 7.6 (104.4 to 116.1) | 0.894 | 0.07 |

*MOWHTO, medial open–wedge high tibial osteotomy; LCWHTO, lateral closed–wedge high tibial osteotomy.*

*Values are presented as mean ± SD (95% CI); the knee flexion angle was defined as the preoperative to postoperative change; the mean ± SD shown is taken from the original analysis. Effect size r was calculated from the Wilcoxon rank–sum test (r = |Z|/√N).*
