# Supplementary material for: Three‐dimensional computed tomography‐based comparison reveals divergent patellar and tibial tubercle behaviour between lateral closed‐wedge and medial open‐wedge high tibial osteotomy
Source: J Exp Orthop. 2026 Aug 6;13(3):e70884. doi: 10.1002/jeo2.70884 (PMC13446280; doi:10.1002/jeo2.70884)
Supplement: Supplementary file 4 [file JEO2-13-e70884-s002.docx]

**Table S2. Knee Injury and Osteoarthritis Outcome Score (KOOS) subscales at 2 years postoperatively**

| **Parameter** | **MOWHTO (n = 9)** | **LCWHTO (n = 9)** | **P value** | ***r*** |
| --- | --- | --- | --- | --- |
| Symptoms | 90.6 ± 9.7 (82.5 to 98.8) | 90.4 ± 13.0 (80.5 to 100.5) | 0.663 | 0.11 |
| Pain | 92.5 ± 6.5 (87.1 to 97.9) | 90.6 ± 11.3 (82.0 to 99.3) | 0.924 | 0.01 |
| Activities of daily living | 95.6 ± 4.8 (91.5 to 99.6) | 95.3 ± 5.6 (90.9 to 99.6) | 0.956 | 0.05 |
| Sports and Recreation | 81.4 ± 13.6 (70.0 to 92.8) | 83.1 ± 12.6 (73.4 to 92.7) | 0.964 | 0.01 |
| Quality of Life | 79.7 ± 14.1 (67.9 to 91.5) | 85.4 ± 14.7 (74.1 to 96.7) | 0.263 | 0.27 |

*MOWHTO, medial open–wedge high tibial osteotomy; LCWHTO, lateral closed–wedge high tibial osteotomy; KOOS, Knee Injury and Osteoarthritis Outcome Score.*

*Values are presented as mean ± SD (95% CI). Effect size r was calculated from the Wilcoxon rank–sum test (r = |Z|/√N).*
